# Acceptability of the Dapivirine Vaginal Ring for HIV-1 Prevention and Association with Adherence in a Phase III Trial

**DOI:** 10.1007/s10461-021-03205-z

**Published:** 2021-03-13

**Authors:** Ashley J. Mayo, Erica N. Browne, Elizabeth T. Montgomery, Kristine Torjesen, Thesla Palanee-Phillips, Nitesha Jeenarain, Linly Seyama, Kubashni Woeber, Ishana Harkoo, Krishnaveni Reddy, Tchangani Tembo, Prisca Mutero, Thelma Tauya, Miria Chitukuta, Brenda Gati Mirembe, Lydia Soto-Torres, Elizabeth R. Brown, Jared M. Baeten, Ariane van der Straten, for the MTN-020/ASPIRE study team, Jared Baeten, Thesla Palanee-Phillips, Elizabeth Brown, Lydia Soto-Torres, Katie Schwartz, Bonus Makanani, Francis Martinson, Linda-Gail Bekker, Vaneshree Govender, Samantha Siva, Zakir Gaffoor, Logashvari Naidoo, Arendevi Pather, Nitesha Jeenarain, Gonasagrie Nair, Flavia Matovu, Nyaradzo Mgodi, Felix Mhlanga

**Affiliations:** 1 FHI 360, Durham, NC USA; 2 Women’s Global Health Imperative, RTI International, Berkeley, CA USA; 3 Wits Reproductive Health and HIV Institute, University of the Witwatersrand, Johannesburg, South Africa; 4 HIV Prevention Research Unit, South African Medical Research Council, Durban, South Africa; 5 College of Medicine–Johns Hopkins Research Project, Queen Elizabeth Central Hospital, Blantyre, Malawi; 6 Centre for AIDS Programme of Research in South Africa, University of KwaZulu-Natal, Durban, South Africa; 7 University of North Carolina Project, Lilongwe, Malawi; 8 University of Zimbabwe Clinical Trials Research Centre (UZ-CTRC), Harare, Zimbabwe; 9 Makerere University - Johns Hopkins University (MU-JHU) Research Collaboration CRS, Kampala, Uganda; 10 National Institute of Allergy and Infectious Diseases, National Institutes of Health, Bethesda, MD USA; 11 Fred Hutchinson Cancer Research Center, Seattle, WA USA; 12 University of Washington, Seattle, WA USA; 13 University of California San Francisco (UCSF), San Francisco, CA USA; 14 Present Address: Gilead Sciences, Foster City, CA USA

**Keywords:** HIV prevention, Acceptability, Adherence, Vaginal ring, Sub-Saharan Africa

## Abstract

We evaluated the acceptability of the 25 mg dapivirine vaginal ring (DVR) as an HIV prevention intervention and its influence on DVR adherence in the MTN-020/ASPIRE phase III trial. Acceptability measures were captured using ACASI at month 3 and end of product use (median 24 months, IQR 15–30). Monthly returned rings were classified as nonadherent if dapivirine release rate was ≤ 0.9 mg/month. Associations between acceptability measures and nonadherence were estimated using Poisson regression models with robust standard errors. At month 3 (N = 2334), 88% reported DVR was comfortable, 80% were unaware of it during daily activities, and 74% never felt it during sex. At exit, 66% were ‘very likely’ to use DVR in the future. Acceptability was found to differ significantly by country across several measures including wearing the ring during sex, during menses, partner acceptability, impact on sexual pleasure and willingness to use the ring in the future. Risk of nonadherence at month 12 was elevated if DVR was felt during sex at month 3 (aRR 1.67, 95% CI 1.26, 2.23). Risk of nonadherence in the last year of study participation was elevated if, at exit, participants minded wearing during sex (aRR 2.08, 95% CI 1.52, 2.85), during menses (aRR 1.57, 95% CI 1.06, 2.32), reported a problematic change to the vaginal environment (aRR 1.57, 95% CI 1.12, 2.21), and were not “very likely” to use DVR in the future (aRR 1.31, 95% CI 1.02, 1.68). DVR acceptability was overall high yet varied by country. Addressing perceived ring interference with sex, menses, or problematic changes to the vaginal environment in future interventions could help improve adherence, as could embracing sex-positive messaging related to ring use and increased pleasure.

*Trial Registration* ClinicalTrials.gov Identifier: NCT01617096.

**Supplementary Information:**

The online version contains supplementary material available at 10.1007/s10461-021-03205-z.

## Introduction

More than half of people living with HIV worldwide are women and those living in sub-Saharan Africa bear a disproportionate burden of new HIV infections each year [1]. They require and desire options for HIV prevention that are safe, effective, and acceptable [2, 3]. The dapivirine vaginal ring (DVR) is one potential option. Two randomized, placebo controlled, phase III trials in healthy sexually-active women, MTN-020/ASPIRE and IPM-027/The Ring Study, demonstrated the monthly DVR was well-tolerated and reduced HIV incidence [4, 5]. In ASPIRE, higher risk reduction was estimated with measures indicating higher adherence [6]. In earlier phase I and II trials the DVR was found to be highly acceptable [7–10].

Assessment of product acceptability is not well standardized and is too often measured as a singular concept. As described by Mensch et al. [11] a framework that evaluates components of acceptability—including dosing regimen, product use attributes, effect of the product on sex, and partner’s attitudes—may help better define the influence of product acceptability on adherence of new HIV prevention methods.

In previous trials, acceptability of vaginal rings was high across several measures, including overall willingness to use in the future (if found effective for HIV prevention) [7, 9], ease of insertion/removal [7, 9, 10], feeling comfortable [7, 9, 10], and being unnoticeable during daily activities or once inserted [7, 9, 10]. Furthermore, early trials indicate most do not feel the ring during sex [7] or that it does not interfere with sex [9]. Per participant report, their male partners were more likely to have felt the ring during sex, however, only a minority reported this to be a problem for continued ring use [7]. Qualitative analysis from ASPIRE indicated that participants generally liked the ring better with experience, that adherence challenges could usually be overcome with staff or peer support, and that male partners were a commonly cited influence on ring acceptability and adherence [12]. The ring also impacted sexual experience in many ways, for some positively and for others negatively [13].

Here we present a quantitative analysis of DVR acceptability in the ASPIRE trial, one of the first cohorts to use the ring for an extended period (up to 3 years). We evaluated dimensions of acceptability, changes throughout the trial, and their independent influence on an objective measure of adherence. Understanding the acceptability of the DVR, the importance of each component, and the potential influence of acceptability on product adherence, is critical for supporting the ring’s successful roll-out and use in sub-Saharan Africa.

## Methods

### Study Population and Design

MTN-020/ASPIRE (NCT01617096) was a phase III randomized, double-blind, placebo-controlled clinical trial conducted across 15 sites in Malawi (two sites), South Africa (nine sites), Uganda (one site), and Zimbabwe (three sites) that enrolled 2629 women aged 18–45 between August 2012 and June 2015. Participants were randomized in a 1:1 ratio to use either a monthly silicone elastomer vaginal matrix ring containing 25 mg of dapivirine or a placebo ring. Participants were counseled on how to insert and remove the ring and were instructed to keep the ring inserted for the entire month. Eight months after trial initiation, several site-specific participant engagement activities and monitoring strategies were implemented to improve adherence and retention in the trial [14]. Study visits occurred monthly, and at each visit, participants received HIV-1 serologic and pregnancy testing, safety monitoring, and individualized adherence counseling based on reported ring use experiences. Participants were followed for a minimum of one year and for a maximum of 33 months. Intention-to-treat analysis showed 27% reduction in HIV incidence [95% confidence interval (CI), 1–46; P = 0.05] in the DVR arm compared to placebo. Additional details of the trial design, recruitment, and results have been published previously [4].

### Measures

#### Acceptability

Acceptability data were captured using audio computer-assisted self-interview (ACASI) at the month-3 visit and at the Product Use End visit (PUEV), the penultimate visit when study product dispensing was permanently discontinued, among participants who self-reported using the ring. Participants were asked during each ACASI to reflect on their experiences using the ring in the prior 3 months. In addition, the PUEV ACASI collected participants’ views of the ring in general, based on their experience throughout the trial. Participants also completed an ACASI at baseline which assessed sexual behavioral characteristics and likelihood of future ring use. Acceptability measures, response options, and frequency of assessment during the trial are summarized in Table 1.

**Table 1:** ASPIRE acceptability measures

Acceptability measure^a^	Response options	Assessed at visit		
		Enrollment	Month 3	PUEV^b^
Overall acceptability				
If in the future a vaginal ring was available that provided some protection against HIV, and it was similar to the one you used in this study, how likely would you be to keep it inserted in your vagina every day?	Very unlikely, unlikely, likely, very likely	X		X
Use attributes				
How difficult was it to insert the vaginal ring the last time you inserted it?	Very difficult, somewhat difficult, not difficult at all		X	X
How difficult was it to take the vaginal ring out the last time you took it out?	Very difficult, somewhat difficult, not difficult at all		X	X
In the past 3 months, how did it feel to have the vaginal ring inside you every day?	Usually comfortable, sometimes uncomfortable, usually uncomfortable		X	X
In the past 3 months, were you aware of the vaginal ring during your normal daily activities?	Most of the time, sometimes, never		X	X
Did you mind wearing the ring during menses?	Yes, no, did not wear the vaginal ring during menses, did not have menses during the study			X
Have you noticed any of the following changes in your vagina while wearing the vaginal ring: vagina was wetter, vaginas was drier?	Vagina was wetter, vagina was drier, no change noticed			X
[If wetter or drier]: was this change a problem for you?	Yes, no			
Effects on sex				
In the past 3 months, how often did you feel the vaginal ring inside you when you had sex?	Most of the time, sometimes, never, did not have sex in the past 3 months		X	X
In the past 3 months, did any of your partners feel the vaginal ring inside of you when you had sex?	Yes, no, don't know		X	X
How does the vaginal ring affect your sexual pleasure?	Increases pleasure, does not change pleasure, decreases pleasure			X
Did you mind wearing the ring during sex?	Yes, no, did not wear the vaginal ring during sex, did not have sex during the study			X
Partner's attitude				
Was the vaginal ring acceptable to your primary partner?	Yes, no, don't know			X
Has your primary sex partner ever asked you to stop wearing the ring?	Yes, no			X

^a^Measures are organized by component of acceptability as outlined by Mensch et al. [11]

^b^*PUEV* product use end visit, the penultimate visit when study product dispensing was permanently discontinued (median 24 months, IQR 15–30 months)

#### Adherence

For this analysis, adherence was determined by residual dapivirine in returned vaginal rings, which were collected monthly starting approximately one calendar year after trial initiation. Acetone extraction and high-pressure liquid chromatography measured residual dapivirine in returned used rings. Previous research has established that with consistent use of the ring in a 28-day period, at least 4 mg of dapivirine is released [15]. From lab measures of unused rings, dapivirine release rates ≤ 0.9 mg per month indicated no or very low use of the ring (the equivalent of one standard deviation of lab measurement error). Because study visits did not occur exactly every 28 days, adherence was based on the ratio of the amount of dapivirine released (25 mg minus the remaining dapivirine) to the number of days since the ring was dispensed. Rings with a release rate equivalent to ≤ 0.9 mg per month were classified as nonadherent [16]. If a participant indicated a ring was lost or did not returned a ring, the ring was assumed to have not been used (i.e., release rate ≤ 0.9 mg/month).

### Analysis

Participants who completed acceptability measures during at least one follow-up ACASI (month 3, PUEV) were included in this analysis. Descriptive characteristics of the analysis sample are presented by country to highlight differences in the study population. Chi-square tests or *t* tests were used to evaluate the differences observed. We used logistic regression models, adjusted for country to assess differences in sociodemographic characteristics between the total enrolled sample and the analytic sample. Acceptability measures were summarized by study visit to evaluate changes over time and by country, as per above. Based on the response distribution and possible positive response bias for the overall acceptability measure, analyses compared “very likely” future use to less than very likely (i.e. “likely”, “unlikely,” or “very unlikely”). To test for differences in acceptability over time, we used a mixed effects logistic regression model for each acceptability measure. Models including fixed effects for country, treatment arm, and total months of follow-up and a random effect for participant. For measures only assessed at PUEV, Chi-square tests were used to compare responses by country. Given younger participants were found to be less adherent in the trial [4], we used logistic regression models to explore if acceptability differed between younger and older participants, adjusted for country, treatment arm, and months of follow-up. Young participants were defined as those age 18–21 years at enrollment.

The adherence portion of the analysis excluded all participants randomized to the placebo ring, given adherence was determined by dapivirine drug concentrations. We estimated how early acceptability of the ring (from the month-3 ACASI) influenced nonadherence at month-12, since most participants had residual dapivirine measures at this visit. We also assessed the relationship between acceptability measures collected only at PUEV and nonadherence within the last year of study participation. Nonadherence was dichotomized and defined by having three nonadherent rings in the last 12 months. Only those with at least 5 rings dispensed in the last year were included. Most (80%) had 12 rings dispensed in their last year in the study; 9% had < 5 rings. As a sensitivity analysis, we refit models only with those who had 12 rings dispensed in the last 12 months and found similar effects. We estimated separate Poisson regression models with robust standard errors for each acceptability measure to determine if it was associated with nonadherence. Models were stratified by age for acceptability measures found to differ by age group. All models controlled for country, months of follow-up, and enrollment post initiation of the adherence monitoring and engagement activities.

All analyses were performed using Stata 15.0 (StataCorp LLC, College Station, TX). P values < 0.05 were considered significant. Local ethical approval was obtained from all study sites prior to trial implementation. Informed consent was obtained from all individual participants included in the study [4].

## Results

Among the 2629 participants enrolled in the ASPIRE trial, 2562 (97%) completed the acceptability measures on at least one follow-up ACASI and were included in the analysis. There were no significant differences between participants included in the analytic sample and those without ACASI who were excluded (all P ≥ 0.09). Descriptive characteristics of the analytic sample are presented in Table 2. Participants across countries differed on almost every characteristic evaluated (Supplemental Table 1). Across all sites, the average age of participants was 27 with 20% considered young (between the ages of 18–21); 46% had completed secondary school, 41% were married, and 45% earned an income of their own. Nearly all (98%) had a primary partner and were parous (92%). At enrollment, 58% had more than one male sex partner in the past 3 months. On average, participants were followed for 21.5 months (median 21, interquartile range 14–29 months).

**Table 2:** Baseline characteristics of participants in the ASPIRE trial (Aug 2012–Jun 2015) who completed acceptability measures at month-3 and/or PUEV

	Total	
	N	(%)
Total	2562	(100)
Age, years—mean, median (IQR)	27.2, 26	(22–31)
18–21	507	(20)
22–45	2055	(80)
Completed secondary school	1172	(46)
Earns own income	1156	(45)
Has primary sex partner	2496	(98)
Currently married	1058	(41)
≥ 2 male sex partners in past 3 mo	1489	(58)
Transactional sex in past year	156	(6)
Parity > 0	2350	(92)
Current contraceptive method		
Injectable	1407	(55)
Implant	494	(19)
Intrauterine device (IUD)	319	(13)
Oral contraceptive pills	278	(11)
Male condoms	94	(4)
Sterilization^a^	77	(3)

*PUEV* product use end visit, *IQR* interquartile range

^a^Tubal ligation/hysterectomy/laparoscopy/other surgical procedure that causes sterilization

### Acceptability

**Fig. 1:**
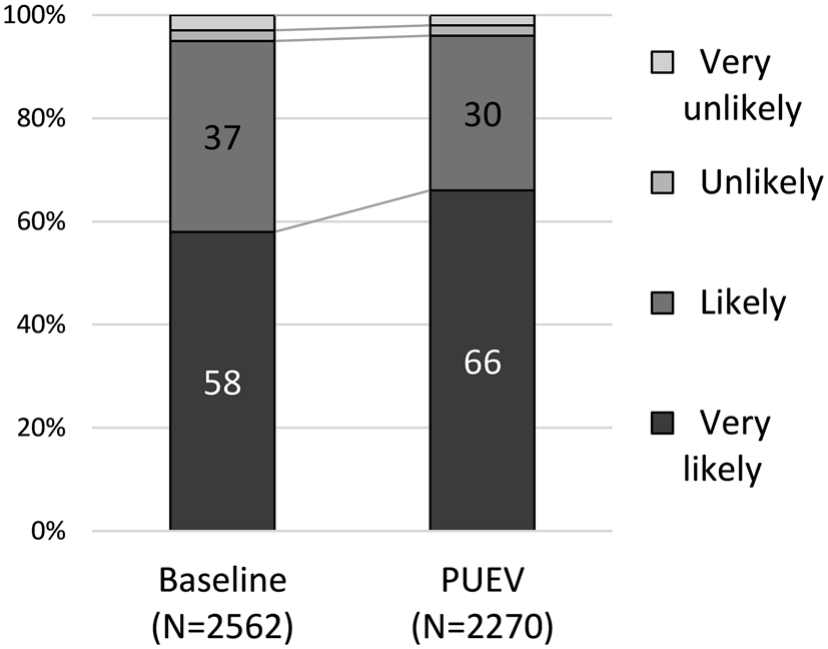
Overall acceptability of the dapivirine vaginal ring, as measured by future likelihood of use at baseline and PUEV

Overall, at PUEV nearly all participants reported they were “very likely” (66%) or “likely” (30%) to use a monthly vaginal ring in the future. More were “very likely” compared to baseline [adjusted odds ratio (AOR): 1.5 95% CI 1.3, 1.7, P < 0.001; Fig. 1). There were significant differences in future willingness to use by country at both baseline and PUEV, with about half of participants in Malawi and South Africa stating they were “very likely” compared to about three-quarters in Uganda and Zimbabwe (Supplemental Table 1, Table 4).

Acceptability of the ring (based on prior 3 months of use) collected at month 3 and PUEV are compared in Table 3. Much of the analytic sample had month 3 ACASI data available (n = 2474, 97%), and among these, 2334 (94%) self-reported using the ring in the past 3 months and provided responses to acceptability measures. In general, the ring was found acceptable across several components of acceptability. Most participants found the ring not difficult to insert (85%) or remove (76%), usually comfortable (88%), and were unaware of it during normal daily activities (80%) or during sex (74%). Sixty-nine percent reported that their partner(s) did not feel the ring during sex and 12% did not know if their partner(s) had felt the ring during sex in the past 3 months.

**Table 3:** Acceptability of the dapivirine vaginal ring based on use in the past 3 months, at month-3 visit and PUEV

	Month 3		PUEV	
	N	(%)	N	(%)
Total who used ring in past 3 months^a^	2334	(100)	2075	(100)
Use attributes: ease and comfort of use				
How difficult was it to insert the ring?^b^				
Very/somewhat difficult	346	(15)	128	(6)
Not difficult at all	1974	(85)	1938	(93)
How difficult was it to take the ring out?^b^				
Very/somewhat difficult	326	(14)	197	(10)
Not difficult at all	1776	(76)	1617	(78)
Never took out ring in past 3 months	232	(10)	261	(13)
Use attributes: physical sensation in situ				
How did it feel to have the ring inside you every day?^b^				
Usually comfortable	2047	(88)	1907	(92)
Sometimes/usually uncomfortable	286	(12)	167	(8)
Were you aware of the ring during normal daily activities?^b^				
Most/some of the time	478	(21)	342	(16)
Never	1855	(80)	1732	(84)
Effects on sex				
Total who had sex in past 3 months	2310	(99)	2011	(97)
How often did you feel the ring during sex?				
Most/some of the time	592	(26)	474	(24)
Never	1716	(74)	1537	(76)
Did any of your partners feel the ring during sex?^b^				
Yes	444	(19)	348	(17)
No	1600	(69)	1389	(69)
Don’t know	265	(12)	273	(14)

*PUEV* product use end visit

^a^A total of 2474 participants had ACASI data available from Month 3, and 2270 from PUEV. PUEV ACASI data were missing from 2 sites due to transmission errors

^b^Significant difference between Month-3 and PUEV (p < 0.05). Differences over time were tested using mixed-effect regression models, controlling for country, treatment arm, and number of months of follow-up

Although 2469 participants completed their PUEV visit for ASPIRE, ACASI data were available for 2270 participants (92%), with missing data largely attributable to transmission errors primarily at two sites. Of the 2270 participants who completed the PUEV ACASI, 2075 (91%) stated they had used the ring within the past 3 months and provided responses to acceptability measures. Compared to month 3, a greater proportion of participants at PUEV found the ring not difficult to insert (93%), usually comfortable (92%), and were unaware of it during normal activities (84%).

In addition, at PUEV, participants were asked about their general opinions of the vaginal ring over the entire study period. Acceptability was found to differ significantly by country (Table 4). Significantly more participants in Malawi (35%) and Uganda (85%) minded wearing the ring during sex compared to South Africans (10%) and Zimbabweans (< 1%). Similarly, more in Malawi (35%) and Uganda (77%) minded wearing the ring during menses than in South Africa (8%) and Zimbabwe (1%). Overall, 75% of those who minded wearing the ring during sex also minded wearing it during menses. Thirty percent (n = 683) noticed a change in the vaginal environment while wearing the ring (either the vagina was wetter (20%) or drier (10%)); 187 (8%) reported this change as problematic. Most participants (58%) reported the ring did not affect sexual pleasure, while 39% stated it increased pleasure. Overall, the majority (71%) said the ring was acceptable to their partner, except in Uganda, where less than half (42%) reported the ring was acceptable to their partner.

**Table 4:** Acceptability of the dapivirine vaginal ring at PUEV, based on all months of use, by country

	Malawi		South Africa		Uganda		Zimbabwe		Total	
	N	(%)	N	(%)	N	(%)	N	(%)	N	(%)
Total	259	(100)	1136	(100)	238	(100)	637	(100)	2270	(100)
PUEV study month—mean, median (IQR)	14.4, 13 (11–16)		20.3, 24 (13–27)		21.9, 22 (19–27)		18.9, 19 (13–24)		19.4, 20 (13–27)	
Use attributes										
Problematic change to vaginal environment^a^	19	(7)	125	(11)	19	(8)	24	(4)	187	(8)
Vagina wetter	13	(5)	109	(10)	13	(5)	20	(3)	155	(7)
Vagina drier	6	(2)	16	(1)	6	(3)	4	(1)	32	(1)
Mind wearing the ring during menses^a^	90	(35)	93	(8)	183	(77)	6	(1)	372	(16)
Did not have menses during the study	24	(9)	147	(13)	20	(8)	26	(4)	217	(10)
Effects on sex										
Mind wearing the ring during sex^a^	90	(35)	109	(10)	203	(85)	2	(< 1)	404	(18)
Did not have sex during the study	4	(2)	13	(1)	0	(0)	1	(< 1)	18	(1)
Ring's effect on sexual pleasure^a,b^										
Increases sexual pleasure	152	(59)	358	(32)	85	(36)	283	(44)	878	(39)
Decreases sexual pleasure	4	(2)	44	(4)	10	(4)	3	(1)	61	(3)
No change to sexual pleasure	99	(38)	719	(63)	143	(60)	350	(55)	1311	(58)
Partner’s attitude										
Ring was acceptable to partner^a^										
Yes	231	(89)	794	(70)	101	(42)	484	(76)	1610	(71)
No	15	(6)	142	(13)	59	(25)	34	(5)	250	(11)
Don’t know	13	(5)	199	(18)	78	(33)	119	(19)	409	(18)
Partner ever asked you to stop wearing the ring^a^	20	(8)	158	(14)	27	(11)	34	(5)	239	(11)
Overall acceptability, future likelihood of ring use^a,c^										
Very likely	149	(58)	673	(59)	185	(78)	496	(78)	1503	(66)
Likely	82	(32)	398	(35)	49	(21)	140	(22)	669	(30)
Unlikely	16	(6)	28	(3)	2	(1)	1	(0)	47	(2)
Very unlikely	12	(5)	36	(3)	2	(1)	0	(0)	50	(2)

*PUEV* product use end visit, *IQR* interquartile range

^a^Significantly different by country, p < 0.001

^b^Missing response: Malawi (N = 4), South Africa (N = 15), Zimbabwe (N = 1)

^c^Missing response: South Africa (N = 1)

Most acceptability measures were similar between younger and older participants apart from their perceptions of their partner’s acceptability; those who were younger were more likely to state the ring was not acceptable to their primary partner (16% vs 10%, AOR 1.9, 95% CI 1.4, 2.7; P < 0.001).

### Acceptability and Nonadherence

Table 5 presents the associations between acceptability and nonadherence among participants who were randomized to use the dapivirine ring. First, we estimated how initial acceptability of the ring, measured at month 3, influenced nonadherence at Month 12 (N = 1058). Sixteen-percent of participants were deemed nonadherent based on the ring from month 12 having little to no evidence of use. Participants were more likely to be nonadherent if they felt the ring during sex in the first three months [adjusted relative risk (aRR) 1.67, 95% CI 1.26, 2.23; P < 0.001] and if they said they had a partner who felt the ring during sex (aRR 1.39, 95% CI 0.99, 1.95; P = 0.06). Participants who were aware of the ring during normal activities in the first 3 months seemed modestly more likely to be nonadherent although these results were inconclusive (aRR 1.24, 95% CI 0.91, 1.69; P = 0.17). Initial difficulty with insertion and experiencing discomfort with wearing the ring during the first three months were not associated with nonadherence.

**Table 5:** Associations between acceptability and nonadherence among women randomized to dapivirine vaginal ring

	N (%)	Nonadherence, at Month 12^a^		
		aRR^b^	95% CI	p value
Acceptability measures from month-3 ACASI (N = 1058)				
Use attributes				
Sometimes/usually uncomfortable to have ring inside every day	136 (13)	1.07	(0.73, 1.56)	0.72
Very/somewhat difficult to insert	150 (14)	1.01	(0.70, 1.46)	0.95
Some/most of the time aware of ring during normal activities	216 (20)	1.24	(0.91, 1.69)	0.17
Effects on sex				
Felt ring during sex	256 (24)	1.67	(1.26, 2.23)	< 0.001
Partner felt ring during sex (yes vs. no)	192 (18)	1.39	(0.99, 1.95)	0.06

	N (%)	Nonadherence, in last year of study^c^		
		aRR^b^	95% CI	p value
Acceptability measures from PUEV ACASI (N = 1042)				
Use attributes				
Problematic change to vaginal environment	87 (8)	1.57	(1.12, 2.21)	0.009
Mind wearing during menses	158 (15)	1.57	(1.06, 2.32)	0.02
Effects on sex				
Mind wearing during sex	178 (17)	2.08	(1.52, 2.85)	< 0.001
Ring’s effect on sexual pleasure^d^				
Increases sexual pleasure	430 (41)	Ref	–	–
No change	582 (56)	1.40	(1.07, 1.83)	0.01
Decreases sexual pleasure	21 (2)	1.68	(0.82, 3.45)	0.16
Partner’s attitude				
Ring acceptable to partner (no vs yes)	104 (10)	1.38	(0.98, 1.96)	0.07
General acceptability				
Less than “very likely” to use in the future	345 (33)	1.31	(1.02, 1.68)	0.03

*ACASI* audio computer-assisted self-interview, *CI* confidence interval, *PUEV* product use end visit

^a^Month 12 ring had dapivirine release rate ≤ 0.9 mg/month (N = 165 of 1058; 16%)

^b^Adjusted relative risk (aRR) estimated using separate Poisson regression models with robust standard errors for each acceptability measure. All models adjusted for country, total months of enrollment, and enrollment post adherence intervention initiation

^c^Three rings in last year of study had dapivirine release rate ≤ 0.9 mg/month (N = 200 of 1042; 19%)

^d^Missing response (N = 9)

Second, we assessed how additional acceptability measures collected at PUEV were associated with nonadherence in the last year of the study (N = 1042); 19% met the definition of nonadherent. Participants who stated they minded wearing the ring during sex were twice as likely to be nonadherent (aRR 2.08, 95% CI 1.52, 2.85; P < 0.001). Those who reported a problematic change to the vaginal environment (aRR 1.57, 95% CI 1.12, 2.21; P = 0.009), who minded wearing the ring during menses (aRR 1.57, 95% CI 1.06, 2.32; P = 0.02), and who were less than “very likely” to use a vaginal ring in the future (aRR 1.31, 95% CI 1.02, 1.68; P = 0.03) also had elevated risk of nonadherence. Risk of nonadherence was also associated with impact on sexual pleasure; those who reported no change (aRR 1.40, 95% CI 1.07, 1.83; P = 0.01) or a decrease in pleasure (aRR 1.68, 95% CI 0.82, 3.45) were more likely to be nonadherent than those who found the ring increased sexual pleasure. Participants who reported that the ring was not acceptable to their partners were somewhat more likely to be nonadherent, although these results were inconclusive (aRR 1.38, 95% CI 0.98, 1.96). As partner acceptability was the only measure found to be different by age, we explored the impact of partner acceptability on nonadherence stratified by age. Those aged 22–45 at enrollment with partners who found the ring unacceptable were more likely to be nonadherent (aRR 1.55, 95% CI 1.03, 2.33; P = 0.04), but partner’s attitude was not associated with nonadherence among younger participants (aRR 1.02, 95% CI 0.52, 2.01; P = 0.96).

## Discussion

Across multiple dimensions of acceptability, the dapivirine vaginal ring was highly acceptable to participants and, from their perspective, was also acceptable to their partners. The majority of participants expressed future likelihood of use. However, components of acceptability varied greatly by country, and certain components of acceptability, like effects on sex, had a significant impact on adherence.

Despite vaginal rings being novel in this population, overall acceptability was high and increased over time as participants gained comfort and familiarity with the ring. These findings are similar to early phase dapivirine ring studies [7–9], and supported by qualitative analysis of ASPIRE data, where participants reported liking the ring more with experience [12]. Without a standardized measurement of acceptability, however, it can be challenging to compare findings across studies. The Mensch model [11] provides a framework to evaluate components of acceptability in the context of clinical trials, which, allowed us to better understand factors that may contribute to overall acceptability and observe that some, but not all, elements of acceptability changed over time. For example, while ease of insertion and general comfort with the ring increased, physically feeling the ring during sex for participants and partners remained relatively stable. By assessing both overall and separate components of acceptability, we gained a deeper understanding of ring acceptability, and demonstrate that even though certain elements of ring use may be less acceptable or may change over time, general acceptability may still be high.

We observed notable differences by country both overall and by individual components of acceptability. Generally, participants in Uganda and Zimbabwe reported higher likelihood of future ring use. Individual elements of acceptability also varied by country. For example, those in Malawi and Uganda minded ring use during sex and during menses, whereas this was not a reported concern among participants from South Africa or Zimbabwe. Furthermore, partner acceptability was much lower in Uganda compared to other countries. It is worth noting that some country-specific acceptability measures may seem incongruent with each other. For example, participants in Malawi and Uganda reported minding wearing the ring during sex more often but were also more likely to report increased sexual pleasure. In Uganda, future likelihood of use was among the highest, but minding ring use during menses or sex was also high. These findings speak to several key points. First, the various components of acceptability are not necessarily mutually exclusive. For example, one may mind wearing the ring during sex with certain partners (who may not know about or approve of ring use) but feel increased pleasure with other partners. Similarly, one may mind wearing the ring during sex or menses, but desire or need for an effective HIV prevention method—and therefore their intention for future use of an effective product—may outweigh this dislike. Alternatively, some participants may have disregarded the consistent use aspect of the willingness of future use variable (“keep [the ring] inserted in your vagina every day”), which may also explain some of these apparent disparate results—lack of cognitive testing of this question is a limitation of our analysis. Ultimately, participants in the study were likely balancing pleasure, protection, inconvenience, and partnerships simultaneously in their decision-making, as they probably do similarly in many aspects of their lives every day, including but not limited to contraception.

Our findings of variability in acceptability by country are supported by other work, which demonstrate that preferences around sex, product use, vaginal practices, and hygiene, differ across and within countries [17, 18]. Relationship dynamics, previously noted in ASPIRE qualitative research to have varied by location [19], may be one factor that contributes to the observed differences in acceptability by country. Our findings speak to the importance of conducting research across a range of settings and populations, as well as emphasize the need for offering choices in HIV prevention methods. Individuals from different countries, cultures and backgrounds have different preferences for HIV prevention products, which has potential to influence their uptake and use.

Given the differences observed in age-stratified efficacy of the dapivirine vaginal ring in ASPIRE [4], we expected to find similar variation in acceptability in younger versus older participants. However, most acceptability measures were similar across ages, apart from younger participants being more likely to report the ring was not acceptable to their primary partner. Although reported partner acceptability varied by age, there was no impact observed on adherence related to this component among younger participants. Younger individuals may be more likely involved in shorter term or more casual relationships, or generally be less comfortable talking to their partners about sexual and reproductive health issues, which could explain lower perceived partner acceptability and/or lack of any observed effect of partner acceptability on adherence in this subpopulation. Our observations may also be limited in that only participants who had access to a ring in the 3 months prior to PUEV were administered acceptability questions—younger participants were less likely to receive a ring during this time, so our findings may underestimate potential effect of partner acceptability on adherence among this group. Nonetheless, these findings highlight that while acceptability may have some influence on adherence, it does not explain age-related differences in observed effectiveness in phase three trials, suggesting that other social and structural factors also contribute to ability to use the ring consistently.

Importantly, although perhaps not unexpectedly, participants with low overall acceptability were less likely to use the ring consistently based on objective adherence measurements. Some attributes of acceptability, including effects on sex, perceived negative change to the vaginal environment, and minding wearing the ring during menses, influenced adherence more strongly, whereas others—comfort and ease of insertion—had no effect. Feeling or minding the ring during sex was the strongest influencer of nonadherence observed at both month 12 and in the last year of the study. This is further supported by our findings of increasing nonadherence among participants who reported no change or decreased sexual pleasure, compared to those who reported increased sexual pleasure with the ring. Our observations are supported by previous qualitative research, which demonstrated that the impact of the ring on sexual experiences was a specific concern that participants felt they had to navigate and that had the potential to impact their adherence [13] and that for some, the ring improved their sexual pleasure for reasons ranging from changes in lubrication to reduced fear of HIV acquisition [12]. Participant report of partner acceptability or their partner feeling the ring during sex had a modest influence on nonadherence, although these results were not statistically significant. A stronger relationship between partner acceptability and adherence was observed among older participants. This trend is supported by other qualitative findings from ASPIRE, where partner perceptions were often cited as a prominent influencer of acceptability and adherence [12] as well as work that looked at the relationship between reported partner support and adherence in ASPIRE [20]. Importantly, perceived partner acceptability does not necessarily equate to partner support, highlighting the importance of exploring these relationships both quantitatively and qualitatively.

Recognizing that acceptability may have some influence on adherence or overall willingness for future use, it is important to acknowledge that actual use (adherence) and intention or willingness to use in the future may not always align. This is especially true when adherence is in the context of a randomized controlled trial (RCT) with a placebo and unknown efficacy of the active product. Future willingness to use a known effective product may be high, even among those who struggled with adherence during the RCT due to challenges with use during sex or other barriers; conversely, a participant with high adherence may not ultimately find a product acceptable. Individuals may have many motivations for joining clinical trials outside of product use, and that these motivations may confound observations between components of acceptability and adherence. Furthermore, other important social and structural factors may also play a role, such as desire to have a child, experience of social harms or intimate partner violence [21], initial worries about the ring [22], perception of HIV risk, or acceptability attributes not measured here, such as product characteristics (size, consistency, smell), dosing regimen, or stigma. That said, understanding what components of acceptability most strongly influence adherence may help identify individuals in need of more support and allow for more targeted counseling during initiation of the ring.

There are a few limitations worth noting in our analysis. First, only participants who received a ring in the past 3 months were asked acceptability questions—meaning that, if those who were lost to follow-up or otherwise not receiving rings (e.g., on product hold) were also more likely to find the ring unacceptable, we may be overestimating acceptability of the ring. However, more than 90% of participants did complete acceptability questions so we anticipate that acceptability was still high despite this. Furthermore, PUEV ACASI data were not available from two sites in South Africa, where overall acceptability was somewhat lower (available data from South African participants reported only 59% ‘very likely’ future use) potentially leading to an underestimate of the strength of association between acceptability and nonadherence. Additionally, we did not evaluate all potential components of acceptability, meaning we could have overlooked other key factors that may or may not influence adherence, or contribute to overall acceptability. As already mentioned, we also only measured some acceptability components at PUEV, meaning our analysis of these factors was retrospective and limited our ability to say what effect those factors had on future ring adherence. Also, collection and testing of used rings for residual drug started approximately one year into the trial. Therefore, we are missing some initial residual drug measurements from participants enrolled earlier which could potentially misclassify their level of adherence. Finally, because the error around measures of residual dapivirine with current methods can be up to 0.5 mg, it is challenging to define high adherence by a certain release rate (provided that also the amount of dapivirine released per month is small, about 4 mg over 28-days of use) [15]. Hence, we focused on nonadherence as the definition is more definitive. When more granular quantitative measures of adherence for the vaginal ring are developed, we may find a different relationship between acceptability components and high adherence.

In conclusion, the DVR was highly acceptable to participants across several dimensions of acceptability, although with country variation. Acceptability of the ring increased over time, demonstrating the DVR’s potential as a prevention product for long-term use. Addressing perceived interference with sex, menses, or problematic changes to the vaginal environment, in future interventions through counseling and skill building could help improve adherence, as could embracing sex-positive messaging related to ring use and increased pleasure. Ultimately, the variability we observed in acceptability of the ring highlight the importance of increased choice in HIV prevention options, as no one method will satisfy the needs of all individuals.

## Supplementary Information

Below is the link to the electronic supplementary material.

Supplementary file 1 (DOCX 34 KB)
