# Supplementary material for: Acceptability of the Dapivirine Vaginal Ring for HIV-1 Prevention and Association with Adherence in a Phase III Trial
Source: AIDS Behav. 2021 Mar 13;25(8):2430–40. doi: 10.1007/s10461-021-03205-z (PMC8222015; doi:10.1007/s10461-021-03205-z)
Supplement: Supplementary file 1 — (DOCX 34 KB) [file 10461_2021_3205_MOESM1_ESM.docx]

**Supplemental Table 1.** Baseline characteristics of participants in the ASPIRE trial (Aug 2012- Jun 2015) who completed acceptability measures at Month-3 and/or PUEV, by country

|  | **Malawi** | | **South Africa** | | **Uganda** | | **Zimbabwe** | | **Total** | |
| --- | --- | --- | --- | --- | --- | --- | --- | --- | --- | --- |
|  | **N** | **(%)** | **N** | **(%)** | **N** | **(%)** | **N** | **(%)** | **N** | **(%)** |
| Total | 268 | (100) | 1374 | (100) | 250 | (100) | 670 | (100) | 2562 | (100) |
| Age, years - mean, median (IQR)^a^ | 28.6, 28 (24-33) | | 25.8, 24 (21-29) | | 28.8, 28 (24-33) | | 29.0, 28 (25-33) | | 27.2, 26 (22-31) | |
| 18-21 | 29 | (11) | 395 | (29) | 23 | (9) | 60 | (9) | 507 | (20) |
| 22-45 | 239 | (89) | 979 | (71) | 227 | (91) | 610 | (91) | 2055 | (80) |
| Completed secondary school^a^ | 33 | (12) | 763 | (56) | 27 | (11) | 349 | (52) | 1172 | (46) |
| Earns own income^a^ | 148 | (55) | 502 | (37) | 207 | (83) | 299 | (45) | 1156 | (45) |
| Has primary sex partner | 262 | (98) | 1333 | (98) | 242 | (97) | 659 | (98) | 2496 | (98) |
| Currently married^a^ | 228 | (85) | 106 | (8) | 166 | (66) | 558 | (83) | 1058 | (41) |
| ≥2 male sex partners in past 3 mo^a^ | 168 | (63) | 784 | (57) | 196 | (78) | 341 | (51) | 1489 | (58) |
| Transactional sex in past year^a^ | 14 | (5) | 84 | (6) | 37 | (15) | 21 | (3) | 156 | (6) |
| Parity >0 ^a^ | 266 | (99) | 1166 | (85) | 248 | (99) | 670 | (100) | 2350 | (92) |
| *Current contraceptive method* |  |  |  |  |  |  |  |  |  |  |
| Injectable^a^ | 112 | (42) | 1009 | (73) | 110 | (44) | 176 | (26) | 1407 | (55) |
| Implant^a^ | 119 | (44) | 19 | (1) | 48 | (19) | 308 | (46) | 494 | (19) |
| Intrauterine device (IUD)^a^ | 6 | (2) | 75 | (6) | 71 | (28) | 167 | (25) | 319 | (13) |
| Oral contraceptive pills^a^ | 6 | (2) | 232 | (17) | 14 | (6) | 26 | (4) | 278 | (11) |
| Male condoms^a^ | 0 | (0) | 90 | (7) | 0 | (0) | 4 | (1) | 94 | (4) |
| Sterilization^a, b^ | 25 | (9) | 42 | (3) | 7 | (3) | 3 | (0) | 77 | (3) |
| If a ring was available that provided some protection against HIV,  how likely would you be to keep it inserted every day?^a^ | | | |  |  |  |  |  |  |  |
| Very likely | 138 | (52) | 695 | (51) | 183 | (73) | 467 | (70) | 1483 | (58) |
| Likely | 83 | (31) | 598 | (44) | 58 | (23) | 199 | (30) | 938 | (37) |
| Unlikely | 25 | (9) | 29 | (2) | 3 | (1) | 1 | (0) | 58 | (2) |
| Very unlikely | 21 | (8) | 34 | (3) | 5 | (2) | 3 | (0) | 63 | (3) |
| (no response) | 1 | (0) | 18 | (1) | 1 | (0) | 0 | (0) | 20 | (1) |
| ^a^ Significantly different by country, p<0.001  ^b^ tubal ligation/hysterectomy/laparoscopy/other surgical procedure that causes sterilization | | | | | | | | | | |
